# Supplementary material for: In vitro reconstitution of branching microtubule nucleation
Source: eLife. 2020 Jan 14;9:e49769. doi: 10.7554/eLife.49769 (PMC6959987; doi:10.7554/eLife.49769)
Supplement: Transparent reporting form [file elife-49769-transrepform.docx]

***eLife’s* transparent reporting form**

We encourage authors to provide detailed information *within their submission* to facilitate the interpretation and replication of experiments. Authors can upload supporting documentation to indicate the use of appropriate reporting guidelines for health-related research (see [EQUATOR Network](http://www.equator-network.org/%20)), life science research (see the [BioSharing Information Resource](https://biosharing.org/)), or the [ARRIVE guidelines](http://www.plosbiology.org/article/info:doi/10.1371/journal.pbio.1000412) for reporting work involving animal research. Where applicable, authors should refer to any relevant reporting standards documents in this form.

If you have any questions, please consult our Journal Policies and/or contact us: [editorial@elifesciences.org](mailto:editorial@elifesciences.org).

**Sample-size estimation**

- You should state whether an appropriate sample size was computed when the study was being designed
- You should state the statistical method of sample size computation and any required assumptions
- If no explicit power analysis was used, you should describe how you decided what sample (replicate) size (number) to use

Please outline where this information can be found within the submission (e.g., sections or figure legends), or explain why this information doesn’t apply to your submission:

We based sample-size estimation on previous cell biological and biochemical studies of a similar nature.

**Replicates**

- You should report how often each experiment was performed
- You should include a definition of biological versus technical replication
- The data obtained should be provided and sufficient information should be provided to indicate the number of independent biological and/or technical replicates
- If you encountered any outliers, you should describe how these were handled
- Criteria for exclusion/inclusion of data should be clearly stated
- High-throughput sequence data should be uploaded before submission, with a private link for reviewers provided (these are available from both GEO and ArrayExpress)

Please outline where this information can be found within the submission (e.g., sections or figure legends), or explain why this information doesn’t apply to your submission:

Embryo extracts used for biochemical assays were the result of multiple >5 individual embryo collections.

The Cl-AP procedure and the estimate of cleavage efficiency is based on over 10 individual purification experiments carried out over the life-time of the project; while the amount of pure Msd1-GFP for each purification was estimated by averaging 6 individual purification experiments.

In vitro MT co-sedimentation assays were carried out in triplicate, each using an individually purified batch of Augmin or γ-TuRC.

In vitro polymerization assays to assess the effect of pure Augmin and γ-TuRC presented in Figure 2A are the summation of 3 individual purifications of each protein complex, undertaken in duplicate wells (6 data points)

In vitro polymerization assays to assess the difference in polymerization between cycling and MG132 purified Augmin and γ-TuRC, presented in Figure 2C are the summation of triplicate experiments.

In vitro polymerization assays to assess the difference in Augmin—dependent polymerization upon addition of competing truncated Augmin subunits (Supplementary Figure S2) are the summation of triplicate experiments.

Fluorescent imaging of MTs under different polymerization conditions was undertaken in triplicate (3 independent purifications of Augmin and γ-TuRC). The imaging relating to inclusion of GMPCPP seeds (Figure 3f, g) was undertaken in duplicate.

All the above are explicitly mentioned in the Materials and Methods, within appropriate sections.

**Statistical reporting**

- Statistical analysis methods should be described and justified
- Raw data should be presented in figures whenever informative to do so (typically when N per group is less than 10)
- For each experiment, you should identify the statistical tests used, exact values of N, definitions of center, methods of multiple test correction, and dispersion and precision measures (e.g., mean, median, SD, SEM, confidence intervals; and, for the major substantive results, a measure of effect size (e.g., Pearson's r, Cohen's d)
- Report exact p-values wherever possible alongside the summary statistics and 95% confidence intervals. These should be reported for all key questions and not only when the p-value is less than 0.05.

Please outline where this information can be found within the submission (e.g., sections or figure legends), or explain why this information doesn’t apply to your submission:

Statistical analysis associated with the MT polymerization curves is explicitly detailed in the Materials and Methods, within the “Statistical Analysis of polymerization curves” sub-section.

(For large datasets, or papers with a very large number of statistical tests, you may upload a single table file with tests, Ns, etc., with reference to sections in the manuscript.)

**Group allocation**

- Indicate how samples were allocated into experimental groups (in the case of clinical studies, please specify allocation to treatment method); if randomization was used, please also state if restricted randomization was applied
- Indicate if masking was used during group allocation, data collection and/or data analysis

Please outline where this information can be found within the submission (e.g., sections or figure legends), or explain why this information doesn’t apply to your submission:

Images were taken from randomly distributed fields of the coverslips using a Leica TCS SP8 confocal laser scanning microscope (Materials and Methods).

**Additional data files (“source data”)**

- We encourage you to upload relevant additional data files, such as numerical data that are represented as a graph in a figure, or as a summary table
- Where provided, these should be in the most useful format, and they can be uploaded as “Source data” files linked to a main figure or table
- Include model definition files including the full list of parameters used
- Include code used for data analysis (e.g., R, MatLab)
- Avoid stating that data files are “available upon request”

Please indicate the figures or tables for which source data files have been provided:

We have all the data as a series of Excel sheets which we would like to pull together into a single file. We do not want to delay review so will provide this if the manuscript is accepted. However, please note that all the data points relating the MT polymerization assays is already present in the Figures (Figure 2; Supplementary Figure 2 and 3) – within the graphs themselves each datapoint is represented as a spot.
